# The LapG protein plays a role in *Pseudomonas aeruginosa* biofilm formation by controlling the presence of the CdrA adhesin on the cell surface

**DOI:** 10.1002/mbo3.301

**Published:** 2015-10-12

**Authors:** Morten Rybtke, Jens Berthelsen, Liang Yang, Niels Høiby, Michael Givskov, Tim Tolker‐Nielsen

**Affiliations:** ^1^ Costerton Biofilm CenterDepartment of Immunology and MicrobiologyFaculty of Health and Medical SciencesUniversity of CopenhagenDK‐2200CopenhagenDenmark; ^2^ Singapore Center on Environmental Life Sciences EngineeringNanyang Technological UniversitySingapore639798Singapore; ^3^ Department of Clinical MicrobiologyRigshospitaletCopenhagenDenmark

**Keywords:** Adhesin, biofilm, c‐di‐GMP, protease, Pseudomonas.

## Abstract

*Pseudomonas aeruginosa* is a clinically relevant species involved in biofilm‐based chronic infections. We provide evidence that the *P. aeruginosa* LapG protein functions as a periplasmic protease that can cleave the protein adhesin CdrA off the cell surface, and thereby plays a role in biofilm formation and biofilm dispersal. The *P. aeruginosa* LapG protein is shown to be a functional homolog of the *Pseudomonas putida* LapG protein which has previously been shown to function as a periplasmic protease that targets the surface adhesin LapA. Transposon mutagenesis and characterization of defined knockout mutants provided evidence that the CdrA adhesin is a target of LapG in *P. aeruginosa*. A *wspF lapG* double mutant was hyper‐aggregating and hyper biofilm forming, whereas a *wspF lapG cdrA* triple mutant lost these phenotypes. In addition, western blot detection of CdrA in culture supernatants and whole‐cell protein fractions showed that CdrA was retained in the whole‐cell protein fraction when LapG was absent, whereas it was found in the culture supernatant when LapG was present. The finding that CdrA is a target of LapG in *P. aeruginosa* is surprising because CdrA has no homology to LapA.

## Introduction

Bacterial biofilms are recognized to be of major importance in medical, industrial, and environmental settings. Biofilms are multicellular clusters of bacterial cells encased in a self‐produced polymeric matrix, and they can be surface adherent or free‐floating. Biofilms are of particular concern in medical settings because biofilm‐based infections show recalcitrance toward the immune system and the currently available antibiotics (Costerton et al. 1999; Ciofu and Tolker‐Nielsen 2010). Understanding the regulatory networks and cellular components that are involved in biofilm formation will aid in devising new treatment strategies to more efficiently cope with these infections.

Biofilm formation is considered to occur as a progression going through phases of attachment, microcolony formation, biofilm maturation, and dispersal (Molin and Tolker‐Nielsen 2003). Biofilm formation is in many bacteria regulated via the nucleotide second messenger c‐di‐GMP (Romling et al. 2013). The c‐di‐GMP molecule is produced by diguanylate cyclases through their GGDEF domain and degraded by phosphodiesterase through their EAL or HD‐GYP domains. In addition, many of these proteins harbor sensory domains allowing the level of c‐di‐GMP to be regulated in response to environmental cues. The c‐di‐GMP secondary messenger stimulates biofilm formation by binding to various effector proteins that in turn affects expression of extracellular biofilm matrix components (Ryan et al. 2012). The biofilm matrix is in most cases composed of exopolysaccharides, extracellular DNA, and proteins (Pamp et al. 2007). Cell surface adhesins, which can function in surface attachment, adherence to the exopolymeric matrix, or cell‐to‐cell adherence, are an important class of biofilm matrix proteins. Examples of cell surface‐associated adhesins include antigen 43 from *Escherichia coli* (Henderson et al. 1997; Heras et al. 2014), Bap from *Staphylococcus aureus* (Cucarella et al. 2001), CdrA from *Pseudomonas aeruginosa* (Borlee et al. 2010), and LapA from *Pseudomonas putida* and *Pseudomonas fluorescens* (Espinosa‐Urgel et al. 2000; Hinsa et al. 2003; Gjermansen et al. 2010).

The LapA protein is essential for biofilm formation by the pseudomonads *P. putida* and *P. fluorescens* (Hinsa et al. 2003; Gjermansen et al. 2010), and regulation of the adhesin is well‐described. LapA is secreted by the Type I secretion pathway via its cognate ABC transporter encoded by *lapB*,* lapC*, and *lapE* (Hinsa et al. 2003). The presence of LapA on the cell surface post secretion is regulated by the periplasmic protease LapG that cleaves LapA N‐terminally and thereby releases it from the outer membrane (Gjermansen et al. 2010; Newell et al. 2011). The activity of LapG is controlled by the inner‐membrane protein LapD which can sequester LapG through its periplasmic domain preventing it from cleaving LapA (Gjermansen et al. 2010; Navarro et al. 2011; Newell et al. 2011). Sequestration of LapG by LapD is dependent on binding of c‐di‐GMP to its non‐catalytic EAL domain (Newell et al. 2009, 2011; Navarro et al. 2011). Cleavage of LapA by LapG is required for starvation‐induced dispersal of *P. putida* biofilms (Gjermansen et al. 2005, 2010), and phosphate limitation‐induced dispersal of *P. fluorescens* biofilms (Monds et al. 2007). Starvation and phosphate limitation activate the *P. putida* phosphodiesterase BifA (Lopez‐Sanchez et al. 2013) and the *P. fluorescens* phosphodiesterase RapA (Monds et al. 2007), respectively, which removes c‐di‐GMP from LapD resulting in activation of LapG for cleavage of LapA (Newell et al. 2011).

Based on protein sequence analysis LapG was described as being part of a family of bacterial transglutaminase‐like cysteine proteinases (Ginalski et al. 2004). Interestingly, the analysis predicted a putative protease from *P. aeruginosa* to belong to the family. *P. aeruginosa* is a model organism for the study of biofilm formation (Harmsen et al. 2010), and a clinically relevant species involved in a range of biofilm‐based infections, including chronic pulmonary infections in people with cystic fibrosis (Tolker‐Nielsen 2014). Knowledge about the regulation of biofilm formation in *P. aeruginosa* and the components involved has accumulated rapidly (Fazli et al. 2014). However, although homologs to the *P. putida* and *P. fluorescens* LapD and LapG proteins have been described as being encoded by the *P. aeruginosa* genome (Navarro et al. 2011), there has been no investigation on the effects of this putative matrix‐regulating protein pair on *P. aeruginosa* biofilm formation.

Here, we present an investigation in *P. aeruginosa* of the presence and activity of a system homologous to the Lap system of *P. putida* and *P. fluorescens*. Through sequence alignment, complementation studies, and analysis of biofilm formation we found that the *P. aeruginosa* genes PA1433 and PA1434 most likely encode functional homologs of LapD and LapG from *P. putida*. We present evidence that LapG from *P. aeruginosa* is involved in the regulation of biofilm formation and cell aggregation which occurs when the intracellular level of c‐di‐GMP is increased. Transposon mutagenesis provided evidence that the adhesin CdrA is the target of LapG. Deletion of the *lapG* gene resulted in clumping and hyperbiofilm formation in a *P. aeruginosa* strain with elevated c‐di‐GMP levels, but if *cdrA* is also deleted clumping and hyperbiofilm formation did not occur. Western blot analysis provided further evidence that CdrA is targeted by LapG and cleaved off the cell surface of *P. aeruginosa*. The finding that LapG is involved in *P. aeruginosa* biofilm formation by controlling the presence of the adhesin CdrA on the cell surface is surprising because CdrA is not homologous to LapA and the current consensus target of LapG‐like proteases.

## Experimental Procedures

### Bacterial strains, plasmids, primers, and growth conditions

The bacterial strains and plasmids most important to this study are listed in Table 1. The full list of strains and plasmids and a list of the primers used can be found in Table S1 and S2, respectively. Unless stated otherwise, *E. coli, P. aeruginosa*, and *P. putida* strains used in this study were all routinely grown in LB medium and on LB agar. *E. coli* and *P. aeruginosa* were incubated at 37°C and *P. putida* at 30°C.

**Table 1:** List of bacterial strains and plasmids used in this study

Strain or plasmid	Relevant genotype and/or characteristics	Reference or source
Strains		
*Pseudomonas aeruginosa* PAO1		
WT	Wild type	Stover et al. (2000)
*lapG*	*lapG* deletion mutant	This study
*lapG cdrA*	*lapG cdrA* double deletion mutant	This study
*Pel*	*pelA* deletion mutant	Rybtke et al. (2012)
*pel lapG*	*pelA* and *lapG* double deletion mutant	This study
*psl*	*pslBCD* deletion mutant	This study
*psl lapG*	*pslBCD* and *lapG* double deletion mutant	This study
*wspF*	*wspF* deletion mutant	Rybtke et al. (2012)
*wspF lapG*	*wspF* and *lapG* double deletion mutant	This study
*wspF cdrA*	*wspF* and *cdrA* double deletion mutant	This study
*wspF lapG cdrA*	*wspF*,* cdrA* and *lapG* triple deletion mutant	This study
*wspF pel*	*wspF* and *pelA* double deletion mutant	Rybtke et al. (2012)
*wspF pel lapG*	*wspF*,* pelA*, and *lapG* deletion mutant	This study
*wspF psl*	*wspF* and *pslBCD* deletion mutant	This study
*wspF psl lapG*	*wspF*,* pslBCD*, and *lapG* triple deletion mutant	This study
*wspF pel psl*	*wspF*,* pelA*, and *pslBCD* triple deletion mutant	Rybtke et al. (2012)
*wspF pel psl lapG*	*wspF*,* pelA*,* pslBCD, and lapG* quadruple deletion mutant	This study
*wspF pel psl cdrA*	*wspF*,* pelA*,* pslBCD, and cdrA* quadruple deletion mutant	This study
*Pseudomonas putida* OUS82		
WT	Wild type	Kiyohara et al. (1994)
*lapD*	*lapD* deletion mutant	Gjermansen et al. (2005)
*lapG*	*lapG* deletion mutant	Gjermansen et al. (2005)
Plasmids		
pBBR1MCS‐5	Broad‐host‐range expression vector, Gm^R^	Kovach et al. (1995)
pPA1433	pBBR1MCS‐5‐based *P. aeruginosa* PAO1 *lapD* expression vector, Gm^R^	This study
pPA1434	pBBR1MCS‐5‐based *P. aeruginosa* PAO1 *lapG* expression vector, Gm^R^	This study
pMJT‐1	pUCP18‐based araC‐P_BAD_ expression vector, Amp^R^/Carb^R^	Kaneko et al. (2007)
pBADcdrAB	pMJT‐1‐based *P. aeruginosa* PAO1 cdrAB expression vector, Amp^R^/Carb^R^	Borlee et al. (2010)
pBT20	TnMariner delivery vector for transposon mutagenesis, Gm^R^ Cm^R^	Kulasekara et al. (2005)

For the full list please see Table S1.

Minimal AB medium (A: 15.1 mmol/L (NH_4_)_2_SO_4_, 33.7 mmol/L Na_2_HPO_4_, 22 mmol/L KH_2_PO_4_, 51.3 mmol/L NaCl; B: 1 mmol/L MgCl_2_, 0.1 mmol/L CaCl_2_) supplemented with 10 *μ*mol/L FeCl_3_ and 10 mmol/L citrate (ABFeC medium) was used as a *P. aeruginosa* selective medium for conjugations involving *E. coli*. Minimal AB medium supplemented with a 10,000‐fold dilution of a trace metal solution (Pamp et al. 2007) and 10 mmol/L citrate (ABTraceC) was used as the medium for *P. aeruginosa* biofilm formation in microplates. P*. putida* biofilm formation in microplates was carried out using LB.

Antibiotic selection, when necessary, was carried out at the following concentrations for *E. coli*: 100 *μ*g ampicillin mL^−1^, 15 *μ*g gentamicin mL^−1^, 25 *μ*g streptomycin mL^−1^, 6 *μ*g chloramphenicol mL^−1^; for *Pseudomonas* sp.: 300 *μ*g carbenicillin mL^−1^, and 60 *μ*g gentamicin mL^−1^.

### Sequence alignments

Amino acid sequences of the *P. aeruginosa* PAO1, *P. putida* KT2440, and *P. fluorescens* Pf0‐1 LapD and LapG homologs were retrieved from the Pseudomonas Genome Database (Winsor et al. 2011). The sequences were aligned using Clustal X ver. 2.1 (Larkin et al. 2007) and shaded for similarity evaluation using BoxShade. Motifs and functionally important residues were marked based on previous phenotypic studies and predictions (Ginalski et al. 2004; Newell et al. 2009; Navarro et al. 2011).

### Knockout vector construction and gene deletion in *P. aeruginosa*

The knockout vectors, pΔlapG and pΔcdrA, used in this study to create defined chromosomal gene deletions of *lapG* and *cdrA*, respectively, were both created using a method developed by Joe J. Harrison (unpubl.). Briefly, in‐frame attB‐flanked deletion cassettes were created using splicing by overlap extension PCR and cloned into the donor vector pDONRPEX18Gm using Gateway BP clonase (Thermo Fisher Scientific, Roskilde, Denmark). The integrity of the deletion cassettes was verified by sequencing.

Defined gene deletions of *lapG* and *cdrA* in *P. aeruginosa* PAO1 and derived mutants were made using the allelic exchange method of Joe J. Harrison (unpubl.). In short, the knockout vectors were introduced into *P. aeruginosa* by triparental mating using the mobilization helper plasmid pRK600, and vector integration by a single cross‐over event were selected for by growth on *P. aeruginosa* selective plates supplemented with gentamicin. Excision of the vector backbone by a second cross‐over event was induced by counter selection on sucrose supplemented (10% w/v) LB agar lacking NaCl at 30°C and confirmed by susceptibility toward gentamicin. Clones harboring the desired deletion genotype were identified by colony PCR. Deletions of *pslBCD* were created essentially as described in Rybtke et al. (2012).

### Expression vector construction and introduction in *P. aeruginosa*

The broad‐host‐range cloning vector pBBR1MCS‐5 was used to create expression vectors of *lapD* and *lapG*. Primers were designed for unidirectional insertion of the coding sequences between the XbaI and HindIII restriction sites of the multiple cloning site. In addition to the restriction site, the forward primers contained an extension providing an optimized ribosomal binding site for efficient translation. The vectors were introduced in *P. aeruginosa* and *P. putida* by electroporation using the standard sucrose‐based method for preparation of competent cells (Choi et al. 2006).

### Transposon mutagenesis

Transposon mutagenesis using the TnMariner delivery vector pBT20 (Kulasekara et al. 2005) was employed for the identification of candidates for LapG‐mediated cleavage. pBT20 was introduced in the *wspF lapG* recipient strain by conjugation in the following way: Outgrown cultures of the pBT20 donor and the *wspF lapG* recipient were mixed in a 1:1 ratio, washed twice with LB, and resuspended in 100 *μ*L of LB. The conjugation mix was then spotted on a cellulose acetate filter situated on an LB plate. Once dried, the plate was incubated for conjugation to take place at 37°C for 5 h. After conjugation, the cells were scraped off the filter and resuspended in 500 *μ*L of saline solution (0.9% w/v). The cell suspension was subsequently plated on selective plates in sufficient dilutions for single colonies to appear and incubated at 37°C. Two independent rounds of conjugation were carried out.

Once visible colonies appeared on the selective plates, the transconjugants were scraped off the plates and resuspended in PUM buffer for an enrichment procedure based on the Bacterial Attachment To Hydrocarbon (BATH) assay as described by Gjermansen et al. (2010). In short, the cell suspension was adjusted to have an optical density at 600 nm of 2, and 2 mL of this suspension was added to a glass tube. Hexadecane of 200 *μ*L was placed on top of the suspension as the hydrophobic phase and the tube was vortexed vigorously for 90 sec to mix the two phases. Upon complete phase separation, the aqueous phase was transferred to a new tube and a new hydrophobic hexadecane layer was added. This enrichment procedure was repeated a total of 16 times for each conjugation event.

Following the enrichment procedure, transconjugants remaining in the aqueous phase were plated on selective plates and a total of 250 randomly chosen colonies for each conjugation event were picked for testing using the liquid culture aggregation assay. The resulting cultures were inspected for their aggregation phenotype and cultures displaying a lack of the parental *wspF lapG* hyper‐aggregating phenotype were subjected to a second round of the aggregation assay. Mutants maintaining their phenotype in the second assay were saved for identification of the transposon integration site.

The site of transposon integration was identified by sequencing of PCR fragments generated by two consecutive rounds of arbitrary PCR. In short, the first round of arbitrary PCR was carried out using freshly grown colonies as DNA template and the primers Rnd1‐Pp1, Rnd1‐Pp2, and TnMGm. For the second round of arbitrary PCR, purified PCR product from the first round was used as template together with the primers Rnd2‐Pp and Rnd2‐TnMGm. Programs for the two rounds of arbitrary PCR can be found in Table S3. The purified products from the second round of PCR were sent for sequencing using the primer TnMSeq. The sequences obtained from the arbitrary PCR were compared against the annotated *P. aeruginosa* PAO1 genome using nucleotide BLAST (Altschul et al. 1990) for identification of the site of transposon insertion.

### Biofilm formation in microplates

Biofilm formation in 96‐well microplates (96F, Techno Plastic Products, Trasadingen, Switzerland) was investigated using a modified version of the method described by O'Toole and Kolter (1998). Outgrown cultures of the tested strains were diluted 100‐fold in ABTraceC medium and an inoculum of 100 *μ*L was used per well. Plates were incubated statically at 37°C for 6 and 20 h for the individual experiments.

For quantification of biofilm levels, the plates were emptied by decanting, washed once with water, and stained with 150 *μ*L of a 0.1% crystal violet solution (Sigma‐Aldrich, Brøndby, Denmark). After two additional washes, the plates were dried and the crystal violet was solubilized by addition of 200 *μ*L acetic acid (30% v/v). Absorbance at 590 nm was used for quantification of the solubilized crystal violet stain.

### Aggregation of liquid cultures

For investigation of aggregation in liquid cultures, round‐bottomed glass tubes (borosilicate, Ø 13 mm) were filled with 2 mL of LB medium and inoculated with a single freshly grown colony. The cultures were incubated in a tilted manner and grown for 18 h at 37°C on a shaking table with an orbital shaking pattern (Ø 30 mm) and a shaking speed of 200 rpm before pictures were taken. Aggregation dependent on *cdrAB* overexpression was investigated using an inoculum of 10 *μ*L from an outgrown culture and an incubation time of 6 h. Expression of *cdrAB* was induced using 1% (w/v) l‐arabinose (Sigma‐Aldrich, Brøndby, Denmark).

### Western blotting

Samples for western blot detection of CdrA were obtained in the following way: Outgrown cultures were diluted 200‐fold in fresh LB medium, and grown until reaching the early logarithmic growth phase. Ten milliliter of culture was sampled and added to an equal volume of protein inhibitor cocktail (Roche Complete Ultra, Hvidovre, Denmark). The samples were separated into supernatant and whole‐cell fractions by pelleting the cells. After sterile filtration the proteins in the supernatant were trichloroacetic acid‐precipitated by upscaling the method described by Mougous et al. (2006). The precipitated supernatant proteins and the whole‐cell pellicles were resuspended in 100 *μ*L and 300 *μ*L, respectively, of Laemmli buffer supplemented with dithiothreitol and boiled at 99°C for 5 min. Protein concentrations were measured using the Pierce 660 nm Protein Assay (Thermo Fisher Scientific) and 5 *μ*g of whole‐cell protein were loaded for SDS_PAGE. The amount of supernatant protein loaded for SDS‐PAGE was determined based on the ratio of total normalized supernatant protein to the corresponding total whole‐cell protein. Following SDS‐PAGE, the separated proteins were blotted onto a PVDF membrane (Immobilon‐P, Millipore, Hellerup, Denmark) and blocked with a 2000‐fold dilution of CdrA specific antibody (Borlee et al. 2010). The blot was visualized using a 10,000‐fold dilution of peroxidase‐conjugated goat anti‐rabbit secondary antibody (Sigma‐Aldrich, Brøndby, Denmark) in combination with a chemiluminescent peroxidase substrate (Immobilon, Millipore).

## Results

### PA1433 and PA1434 encode functional homologs of LapD and LapG from *P. putida*

**Figure 1:**
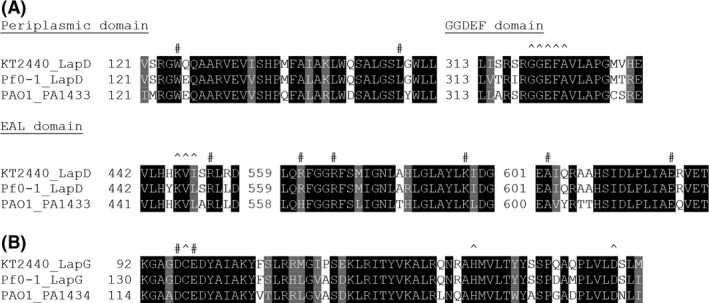
Partial alignments of the amino acid sequences of the LapD (A) and LapG (B) homologs from *Pseudomonas putida* KT2440 (PP0165 and PP0164), *Pseudomonas fluorescens* Pf0‐1 (Pfl01_0131 and Pfl01_0130), and *Pseudomonas aeruginosa* PAO1 (PA1433 and PA1434). (A) ^Degenerate GGDEF and EAL motifs. #Functionally important residues as described by Newell et al. (2009), and Navarro et al. (2011). (B) ^The catalytic triad as predicted by Ginalski et al. (2004). #Functionally important calcium binding residues as described by Boyd et al. (2014). Black and gray shading denotes identical and similar residues, respectively, across all three sequences. Complete alignments are available as Figures S1, S2.

The involvement of the Lap proteins in biofilm formation and dispersal by *P. putida* (Gjermansen et al. 2005, 2010), and the indication that a LapG homolog is encoded by *P. aeruginosa* (Ginalski et al. 2004), made it of interest to us to investigate the putative presence and function of a similar set of proteins in *P. aeruginosa*. Protein BLAST against the annotated *P. aeruginosa* PAO1 proteome using the amino acid sequences of *P. putida* KT2440 LapD, LapG, and LapA indicated that the genes *PA1433* and *PA1434* encode proteins with a high degree of similarity to LapD and LapG, respectively. However, the BLAST search did not identify significant similarity between *P. putida* LapA and any protein encoded by *P. aeruginosa* PAO1. Comparing *lapD* and *lapG* to *PA1433* and *PA1434*, respectively, we noted that the two genes were predicted to form an operon in both *P. putida* and *P. aeruginosa* and that the encoded proteins were predicted to share the same domain organization (Winsor et al. 2011). Furthermore, alignment of the LapD and LapG homologs from *P. putida* and *P. fluorescens* together with the amino acid sequences encoded by *PA1433* and *PA1434* from *P. aeruginosa* revealed a very high degree of sequence conservation for both proteins across all three species (Fig. 1 and Figs. S1, S2). In addition, residues demonstrated experimentally to be functionally important for the two proteins (Ginalski et al. 2004; Newell et al. 2009; Navarro et al. 2011; Boyd et al. 2014) were fully conserved. This includes residues of LapD involved in LapG retention and binding of c‐di‐GMP (Fig. 1A), and residues of LapG involved in calcium binding required for enzymatic activity against LapA (Fig. 1B). Taken together, the bioinformatic analyses made *PA1433* and *PA1434* good candidates for encoding functional homologs of LapD and LapG, respectively.

**Figure 2:**
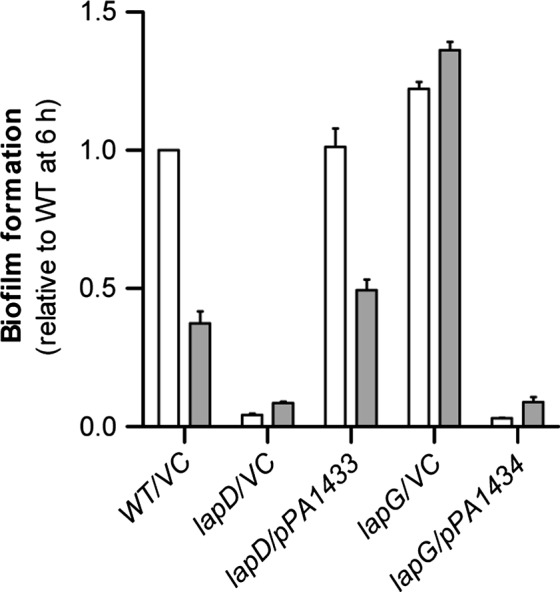
Biofilm formation in microplates by *Pseudomonas putida* OUS82 *lapD and lapG* mutants complemented with their putative functional homologs from *Pseudomonas aeruginosa* PAO1. Data are from biofilm formation after 6 h (white bars) and 20 h (gray bars). Data are presented as values relative to biofilm formation by the WT at 6 h and represent averages of 6 replicates. Error bars indicate standard deviation. VC, vector control.

As no functional homolog of LapA appeared to be present in *P. aeruginosa* PAO1 the putative function of *PA1433* and *PA1434* was initially assessed by introducing the genes *in trans* in *P. putida* OUS82 *lapD* and *lapG* deletion mutants, respectively. *P. putida lapD* mutants have previously been shown to be deficient in biofilm formation because LapD is required for repression of the proteolytic activity of LapG (Gjermansen et al. 2010). Moreover, studies with microplate biofilms have demonstrated that the *P. putida* wild type (WT) and *lapG* mutant form the same amount of biofilm up to a certain level where the WT biofilm biomass decreases due to starvation‐induced biofilm dispersal, whereas the *P. putida lapG* biofilm biomass does not decrease (Gjermansen et al. 2010). Using the microplate biofilm as model system, *PA1433* was shown to complement the biofilm defect of a *P. putida lapD* deletion mutant (Fig. 2). Similarly, *PA1434* was shown to revert the nondispersing/hyper‐biofilm forming phenotype of a *P. putida lapG* deletion mutant (Fig. 2). Overexpression of *PA1434* from the plasmid likely resulted in excess levels of PA1434 unable to be fully controlled by LapD, and resulting in a phenotype resembling the phenotype observed for the *lapD* mutant where LapG activity is de‐regulated. Together with the sequence analysis, these results strongly indicate that *PA1433* and *PA1434* encode functional homologs of LapD and LapG, respectively, in *P. aeruginosa* PAO1. We therefore use the *lapG* and *lapD* designations for the *P. aeruginosa* genes in the following text.

### lapG is involved in the regulation of *P. aeruginosa* biofilm formation in microplates

**Figure 3:**
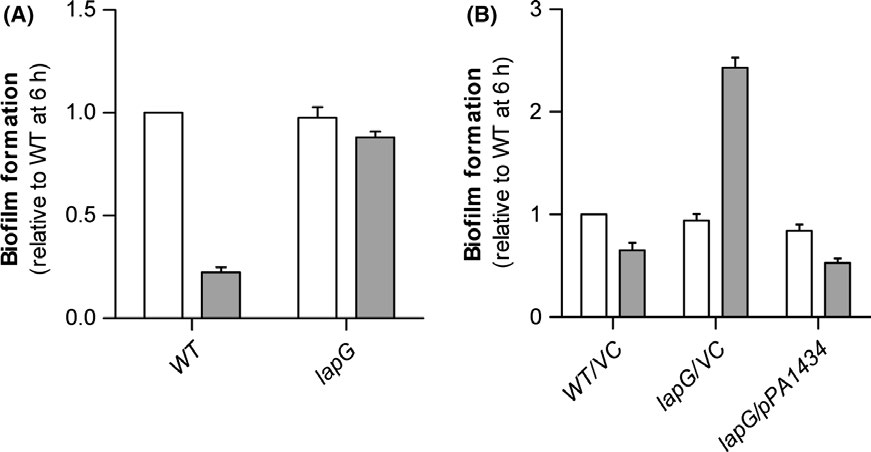
Biofilm formation in microplates by *Pseudomonas aeruginosa* PAO1. (A) *lapG* deletion analysis. (B) Complementation analysis of the *lapG* deletion. Data are from biofilm formation after 6 h (white bars) and 20 h (gray bars). Data are presented as values relative to biofilm formation by the WT at 6 h and represent averages of six replicates. Error bars indicate standard deviation. VC, vector control.

With the evidence that LapD and LapG from *P. aeruginosa* PAO1 are functional homologs of LapD and LapG from *P. putida*, we found it of interest to investigate the more precise function of the proteins in *P. aeruginosa*. In this study, we focus on the role of LapG. A defined deletion of *lapG* was created in *P. aeruginosa* PAO1 and the ability of the strain to form biofilms in microplates was investigated (Fig. 3A). After 6 h of incubation the *lapG* mutant displayed a biofilm level similar to the WT. In contrast, after 20 h of incubation the biofilm level for the WT had decreased whereas the level for the *lapG* mutant had not. The increased stability during prolonged incubation could be specifically attributed to *lapG* as complementation of the mutation restored the biofilm levels of prolonged incubation to that of the WT (Fig. 3B). The increased difference in biofilm formation between the early and late time points for the *lapG* mutant harboring the vector control (Fig. 3B) compared to the plasmid‐free mutant strain (Fig. 3A) is likely due to slightly altered growth kinetics between the strains with and without a plasmid. This resulted in a lower absolute level of biofilm formation after 6 h for the strains harboring a plasmid but not after 20 h where the absolute level of biofilm formation was similar irrespective of the presence of a plasmid. The decrease in biofilm formation by the PAO1 WT during prolonged incubation indicates that a dispersal process has taken place at this time point and thus suggests that *lapG*, as found for *P. putida*, is involved in the dispersal process of *P. aeruginosa* PAO1, probably through targeting of a surface protein that affects stability of the biofilm.

### lapG is involved in aggregation when levels of c‐di‐GMP are increased

**Figure 4:**
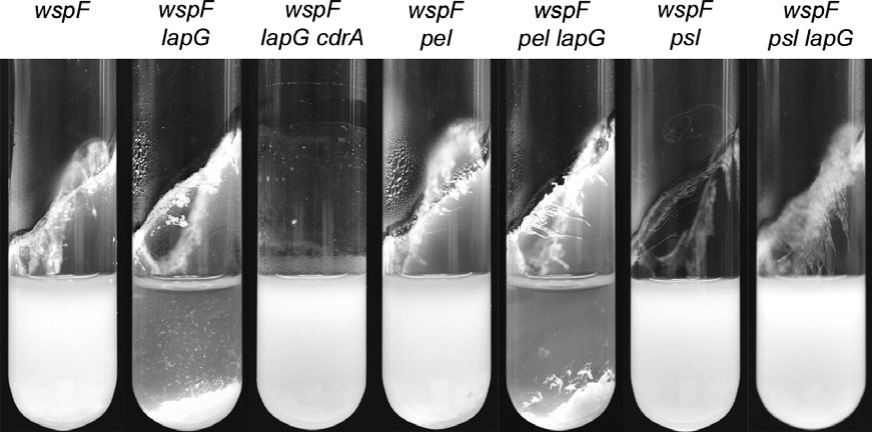
Aggregation phenotype of a *wspF* deletion mutant and derivatives in liquid cultures. Images are of outgrown cultures in glass tubes.

As no LapA homolog is encoded by the *P. aeruginosa* PAO1 genome the target of LapG remained elusive. We therefore considered pursuing identification of the target through TnMariner‐based transposon mutagenesis (Kulasekara et al. 2005). For this, we needed a strong *lapG* deletion phenotype for efficient screening and selection of suppressor mutants. Besides the effect on biofilm dispersal in microplates no phenotype was immediately available for the *lapG* mutant. We hypothesized that levels of the *lapG* target could be regulated by c‐di‐GMP, and therefore working in a strain background with an increased level of the second messenger might uncover useful phenotypes. Therefore, we investigated the effects of deleting *lapG* in a *wspF* deletion mutant which has highly increased c‐di‐GMP levels because of constitutive activation of the diguanylate cyclase WspR (Hickman et al. 2005). The joint *wspF lapG* deletion resulted in a very clear hyper‐aggregating phenotype compared to the parental *wspF* deletion mutant when the strains were grown in liquid cultures (Fig. 4). The *wspF* mutant strain is considered to be hyper‐aggregating but as shown in Figure 4 it also produces a significant planktonic phase indicated by the turbidity of the culture. However, when *lapG* was deleted in addition to *wspF* the planktonic phase almost disappeared completely with the bacteria apparently existing solely as aggregates falling to the bottom of the culture. This observation indicates that LapG is targeting a protein that is involved in cellular aggregation when the level of c‐di‐GMP is increased.

### CdrA is a potential target of lapG

The hyper‐aggregating phenotype of the *wspF lapG* double deletion mutant is highly distinct when grown as liquid cultures in glass tubes and was thus suitable as the phenotypic marker for investigation of the target for LapG. Transposon mutagenesis carried out in the double deletion mutant would identify LapG target candidates from their loss of the hyper‐aggregating phenotype. We employed an enrichment step that separates the transposon mutants based on their cell surface hydrophobicity using a hexadecane/aqueous phase mixture. This enrichment procedure was successfully employed by us in the identification of LapA as a target of LapG in *P. putida* as the adhesin affects cell surface hydrophobicity when present on the surface of the cells (Gjermansen et al. 2010). Because the target of LapG most likely would be a surface adhesin too, we speculated that it would have an effect similar to *P. putida* LapA and increase cell surface hydrophobicity when present on the surface in excess amounts in the *wspF lapG* double deletion mutant. The transposon mutants of interest would thus display a decreased cell surface hydrophobicity and remain in the aqueous phase.

Two separate mutagenesis events were carried out with multiple rounds of subsequent enrichment of cells remaining in the hydrophilic aqueous phase of hexadecane/water containing tubes. From each of the two mutagenesis events, 250 colonies from the aqueous phase of the subsequent enrichment were picked randomly and inoculated for liquid culturing in glass tubes. A total of 18 transposon mutants (15 unique mutants) yielding cultures with a turbid planktonic phase reminiscent of the *wspF* deletion mutant were picked and sequenced for their site of transposon insertion (Table 2, Fig. S3).

**Table 2:** List of transposon mutants in the *wspF lapG* double deletion background identified as deficient in the hyper‐aggregating phenotype of the parent strain

Mutant	Gene
1‐7, 2‐59	*cdrA*
2‐42	*cdrB*
1‐6	*pslA*
2‐54	*pslD*
1‐11	*ladS*
2‐6	*fleN*
1‐3, 1‐5	*wspA*
2‐4	*wspD*
1‐13	*wspR*
2‐3	PA2439/40 intergenic region
1‐1	*cupA3*
2‐2, 2‐40	*pilY1*

What immediately caught our attention were the mutants disrupted in *cdrA* and *cdrB*. *cdrA* has been shown to encode a large surface adhesin involved in biofilm formation by *P. aeruginosa* through tethering of the cells to the Psl exopolysaccharide (Borlee et al. 2010). CdrA and CdrB belong to the Two Partner Secretion family (TCS or Type Vb secretion) with *cdrB* encoding the translocator protein required for transport of CdrA across the outer membrane (Borlee et al. 2010; Leo et al. 2012). The identification of the CdrA adhesin as the likely target of LapG is in line with the hypothesis of LapG serving as a periplasmic protease that regulates the presence of a protein on the surface of *P. aeruginosa*. Corroborating the finding, expression of *cdrA* has been shown to be positively regulated by c‐di‐GMP (Hickman et al. 2005; Hickman and Harwood 2008; Borlee et al. 2010). Also, c‐di‐GMP‐independent overexpression of the *cdrAB* operon results in hyper‐aggregation of *P. aeruginosa* when grown in liquid cultures in a manner similar to the observed hyper‐aggregation of the *wspF lapG* double deletion mutant (Borlee et al. 2010).

### CdrA and Psl polysaccharide are required for aggregation and hyper biofilm formation of *P. aeruginosa* lapG mutants

To verify the findings from the transposon mutagenesis, a defined deletion of *cdrA* was introduced in the *wspF lapG* double deletion mutant, and the effect of the deletion on the aggregation phenotype was investigated (Fig. 4). As expected, deletion of *cdrA* resulted in a loss of the hyper‐aggregating phenotype of the *wspF lapG* mutant*,* thereby confirming the results from the transposon mutagenesis.

Further evidence of CdrA being the target of LapG, came from deletion of *lapG* in *wspF* mutant strains deficient in either Psl or Pel polysaccharide production. As shown in Figure 4, we found that the hyper‐aggregating phenotype resulting from a deletion of *lapG* was dependent upon Psl, but not Pel, polysaccharide production. This observation supports the contention that CdrA is the target of LapG due to the previously mentioned association of CdrA with Psl, but not Pel, polysaccharide (Borlee et al. 2010).

**Figure 5:**
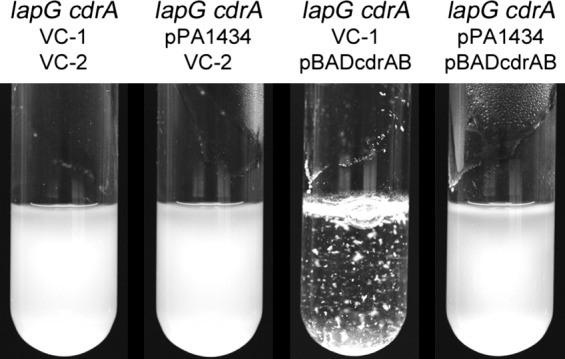
Aggregation phenotypes of *Pseudomonas aeruginosa* strains in liquid cultures in response to overexpression of *cdrAB and lapG*. Overexpression of *cdrAB* and *lapG* was achieved from the pBADcdrAB and pPA1434 plasmids, respectively, whereas the pBBR1MCS‐5 (VC‐1) and pMJT‐1 (VC‐2) plasmids served as vector controls. Images are of cultures induced with 1% (w/v) L‐arabinose grown for 6 h in glass tubes.

In addition to the observations made using the *wspF* strain background with increased levels of c‐di‐GMP we investigated aggregation based on c‐di‐GMP‐independent overexpression of *cdrA* and its transporter *cdrB*. Overexpression of *cdrAB* was found to cause aggregation in the absence of *lapG* (Fig. 5). However, co‐overexpression of *lapG* abolished aggregation (Fig. 5), providing additional evidence for a role of *lapG* in modulating c*drA* dependent phenotypes.

**Figure 6:**
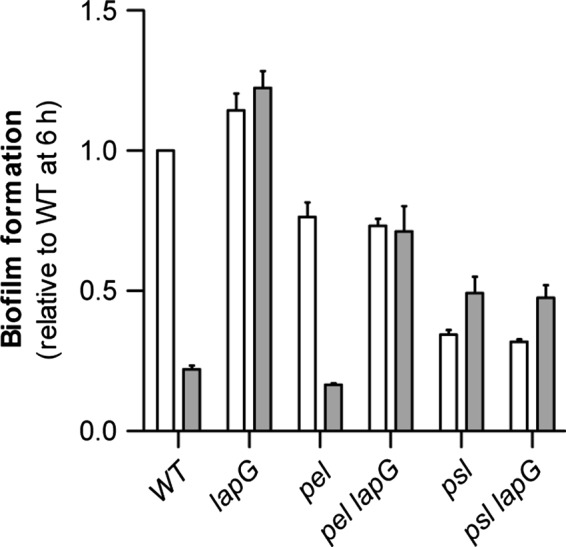
Biofilm formation in microplates by *Pseudomonas aeruginosa* PAO1 polysaccharide and lapG mutants. Analysis of polysaccharide involvement in the *lapG* deletion phenotype. Data are from biofilm formation after 6 h (white bars) and 20 h (gray bars). Data are presented as values relative to biofilm formation by the WT at 6 h, and represent averages of six replicates. Error bars indicate standard deviation.

The involvement of polysaccharides was analyzed further by constructing *psl lapG* and *pel lapG* mutants in the PAO1 WT background. Using this set of mutants we observed that the increased level of biofilm formation seen for the *lapG* mutant after prolonged incubation was dependent upon the presence of the Psl polysaccharide (Fig. 6).

Together with the observations on aggregation in liquid cultures, these observations suggests that LapG targets CdrA and that the activity is required in the dispersal process when Psl polysaccharide is present as part of the biofilm matrix. The thin biofilm formed by the *psl* mutant strain apparently does not undergo dispersal even when an intact *lapG* gene is present.

### Western blot analysis indicates that CdrA is targeted by LapG and cleaved off the cell surface

**Figure 7:**
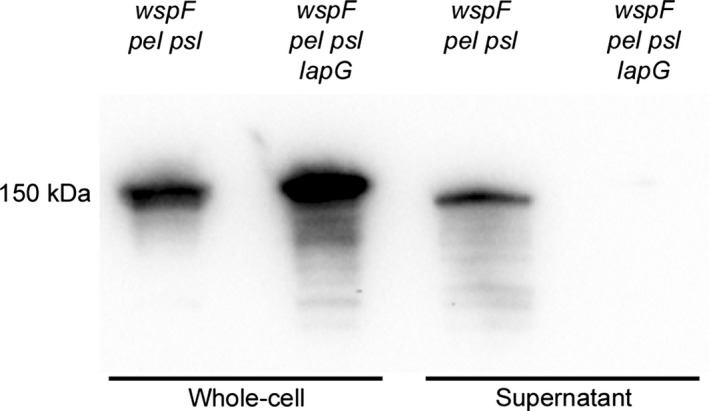
Western blot detection of CdrA. Strain names are listed above the blot and the protein fractions are listed below. Samples were acquired from planktonic cultures during the early logarithmic growth phase.

Additional evidence for CdrA being the target of LapG came from western blot analysis of the spatial presence of the surface adhesin during liquid culturing. A *wspF pel psl* mutant background was chosen for the investigation as previous work has shown that CdrA is only detectable in planktonic cultures when expression is increased by increasing the levels of c‐di‐GMP (Borlee et al. 2010), and because a non‐clumping exopolysaccharide deficient strain ensured ease of planktonic culturing. Anti‐CdrA antibody was used to detect CdrA in whole‐cell and supernatant protein fractions as a band of approximately 150 kDa, as observed previously by Borlee et al. (2010). The antibody proved to be highly specific towards CdrA based on the absence of detectable signal in samples obtained from a *cdrA* mutant (data not shown). For the LapG proficient *wspF pel psl* strain CdrA could be detected in both the whole‐cell and supernatant fraction (Fig. 7). However, in the case of the LapG deficient *wspF pel psl lapG* strain CdrA was evidently retained in the whole‐cell fraction, as an increased amount of CdrA was detected in the whole‐cell fraction and no CdrA was detected in the supernatant fraction (Fig. 7). These results add significant evidence to the contention that CdrA is the target of LapG, and indicate that LapG is involved in cleaving CdrA off the cell surface with a concomitant release of the protein into the surrounding liquid.

## Discussion

In this study, we have established a functional homology between LapD and LapG from *P. putida* and the corresponding proteins encoded by the genes PA1433 (*lapD*) and PA1434 (*lapG*), respectively, in *P. aeruginosa*. Sequence alignment of LapD and LapG from *P. aeruginosa*,* P. putida* and *P. fluorescens* revealed a high degree of sequence conservation across the three species, including functionally important residues for both proteins. Expression of the *P. aeruginosa* genes in *P. putida* complemented the defects in biofilm formation and dispersal of *lapD* and *lapG* deletion mutants, respectively. Together, these observations indicate that the system is conserved across the two species and that LapG has preserved its ability to recognize and cleave the surface adhesin LapA despite the absence of a LapA homolog in *P. aeruginosa*. A putative function of the LapD and LapG regulatory system in *P. aeruginosa* was established based on the result of microplate biofilm formation by *P. aeruginosa* PAO1 and a *lapG* deletion mutant. Looking at initial biofilm formation after 6 h and biofilm formation after prolonged incubation for 20 h we observed that the levels for the PAO1 WT were highest at the early time point and then decreased to a lower level after prolonged incubation. For the *lapG* mutant the levels were similar between the two time points. The biofilm likely experiences nutrient limitation or other unfavorable conditions during the prolonged incubation which leads to biofilm dispersal. The maintained biofilm level observed for the *lapG* deletion mutant after 20 h suggests that LapG is involved in this programmed dispersal much like the case is for LapG from *P. putida* (Gjermansen et al. 2005; 2010). This hypothesis is corroborated by a previous study where evidence was presented that LapG is involved in starvation‐induced dispersal of *P. aeruginosa* biofilms grown in continued culture flow cells (Huynh et al. 2012).

The lack of a *lapA* homolog in *P. aeruginosa* left us with no obvious target for cleavage by LapG. In the search for this target we discovered that increasing the level of c‐di‐GMP had a dramatic effect when *lapG* was also deleted. An increase in the level of c‐di‐GMP through a *wspF* mutation results in a phenotype that among other things is characterized by visible aggregation of cells in liquid cultures due to the overproduction of biofilm matrix components. The cells do, however, continue to form a significant turbid planktonic phase. Deletion of *lapG* in a *wspF* mutant resulted in a clear intensification of the cellular aggregation leading to a significant reduction in turbidity of the planktonic phase with the cellular aggregates collecting at the bottom of the growth vessels.

From a transposon mutagenesis screen based on the hyper‐aggregation phenotype of a *lapG wspF* mutant we isolated three mutants with disruptions of the c‐di‐GMP regulated *cdrAB* operon. *cdrA* encodes a surface adhesin and *cdrB* encodes the CdrA translocator for transport of the adhesin across the outer membrane. Our interest in *cdrA* was further increased by the isolation of *pslA* and *pslD* mutants disrupted in Psl polysaccharide production. CdrA has been shown to be involved in tethering of the biofilm cells to the Psl polysaccharide for increased stability of the exopolymeric matrix (Borlee et al. 2010). In addition, we isolated mutants of *ladS* and *fleN*. LadS is involved in positive regulation of Psl polysaccharide production through activation of the Gac/Rsm regulatory system (Ventre et al. 2006; Irie et al. 2010) and FleN is involved in regulation of *cdrAB* transcription via association with the c‐di‐GMP binding transcriptional regulator FleQ (Hickman and Harwood 2008). We also isolated mutants disrupted in the Wsp signaling cascade, which was expected due to their involvement in the overproduction of c‐di‐GMP through increased activation of the diguanylate cyclase WspR in the *wspF* deletion background.

In addition, we also identified mutants that did not immediately point towards CdrA as the target of LapG. The mutants were disrupted in *cupA3*,* pilY1*, and the PA2439/PA2440 intergenic region. CupA fimbriae are involved in biofilm formation by *P. aeruginosa* PAK (Vallet et al. 2001) and have been shown to be involved in SDS‐induced aggregation of *P. aeruginosa* PAO1 (Klebensberger et al. 2009). *pilY1* encodes a protein involved in type IV pilus biogenesis and is involved in motility and surface adhesion of *P. aeruginosa* (Kuchma et al. 2010). PA2439 encodes a hypothetical protein of unknown function. Interestingly, PA2440 encodes a putative exopolymeric matrix modifying protein, and expression of the gene has been shown to be positively regulated by c‐di‐GMP (Hickman et al. 2005). Work characterizing involvement of the gene in modification of the *P. aeruginosa* biofilm exopolymeric matrix is currently underway in our laboratory.

The combined set of aggregation negative transposon mutants affected in CdrA and Psl polysaccharide production made CdrA a strong candidate as the target of LapG. In accordance, a defined *cdrA* knockout in the *wspF lapG* deletion mutant confirmed the results of the transposon mutagenesis. No hyper‐aggregation was observed in the strain lacking *cdrA* in addition to *wspF and lapG*. In addition, overexpression of *cdrAB* resulted in c‐di‐GMP independent aggregation, whereas simultaneous overexpression of *lapG* prevented aggregation. Corroborating the results, we showed that the *cdrA* dependent phenotypes caused by deletion of *lapG* under both normal and increased levels of c‐di‐GMP required the Psl polysaccharide to which CdrA associates.

In addition to the genetic evidence we also presented biochemical evidence that CdrA is the target of LapG. Western blot detection of CdrA in culture supernatants and whole‐cell protein fractions during liquid culturing showed that CdrA was present solely in the whole‐cell protein fraction when LapG was absent, whereas it was also found in the culture supernatant when LapG was present. This provided further evidence that cleavage by LapG results in release of the CdrA adhesin into the supernatant.

The strong evidence that CdrA is the target for LapG is surprising as this surface adhesin has no apparent homology to LapA, which is the substrate for LapG in *P. putida* and *P. fluorescens*. In addition, the two adhesins are transported across the outer membrane in fundamentally different ways: LapA by its cognate ABC transporter LapBCE, and CdrA by its cognate translocator CdrB. It is interesting to note that LapA is transported in a C to N terminal fashion while the opposite is likely the case for CdrA (Borlee et al. 2010; Newell et al. 2011). In *P. fluorescens*, LapG has been shown to cleave LapA between double alanine residues found several times within a motif present in the N‐terminal part of the protein (Newell et al. 2011; Boyd et al. 2014). It can be speculated that this apparent promiscuity of the active site in LapG enables it to recognize and cleave non‐LapA‐like adhesins, as is the case for CdrA in *P. aeruginosa,* while retaining its ability to cleave LapA. It will be interesting to identify LapG targets in other more distantly related bacterial species where a homolog of LapG, but not LapA, is present.

We looked for a potential LapG cleavage site in the annotated protein sequence of CdrA. With transport of CdrA most likely occurring in the N‐ to C‐terminal direction we scrutinized the C‐terminal end of the protein and indeed found a region of slight homology to the consensus motif of LapA including the pair of alanine residues 2105 and 2106 that might serve as the LapG cleavage site. Our western blot analysis indicated that CdrA detected in the supernatant sample for the LapG proficient strain was a bit smaller in size than the CdrA detected in the whole‐cell samples. This could be the result of cleavage of CdrA by LapG between the alanine residues 2105 and 2106 which would decrease the protein size with about 5 kDa. This is merely speculative, however, and more extensive analysis is required for the proper identification of the exact LapG cleavage site in CdrA.

Our identification of CdrA as the target for LapG‐mediated cleavage in *P. aeruginosa*, and the results from the microplate biofilm formations suggests that LapG and most likely LapD are involved in facilitating dispersal of *P. aeruginosa* biofilms. Together with the results from recent studies implicating a range of phosphodiesterases in the dispersal process of *P. aeruginosa* (An et al. 2010; Roy et al. 2012; Li et al. 2013), and one indicating that LapD is binding c‐di‐GMP (Duvel et al. 2012), we propose that the LapG and LapD pair of proteins functions as an effector system for dispersal induced by phosphodiesterase activation and lowering of the c‐di‐GMP level much like the case is for the system in *P. putida*. The effect is mediated through cleavage of CdrA which has been shown to tether the cells to the Psl polysaccharide resulting in increased structural stability of the biofilm matrix (Borlee et al. 2010). It can be speculated that the structural stability of the matrix is weakened once CdrA is cleaved off the cell surface by LapG which loosens the ties of the cells to the matrix and allows them to disperse more easily.

In a clinical perspective, two studies have shown *cdrA* to be present in clinical isolates from cystic fibrosis patients (Wolfgang et al. 2003; Borlee et al. 2010), suggesting a role of the adhesin for some isolates during this biofilm‐based infection, which is interesting in the context of LapG and the putative role of this protein during infection. The putative role of CdrA and LapG during infection is strengthened further by the implication of a role of Psl polysaccharide in the stability of biofilms formed by alginate overproducing mucoid isolates, which are abundant in cystic fibrosis infections (Yang et al. 2012). Our future studies will aim at deducing the entire signaling pathway from sensing of dispersal‐inducing environmental cues, to induction of LapG activity and cleavage of CdrA.

## Conflict of Interest

None declared.

## Supporting information


**Table S1.** Full list of strains and plasmids used in this study.
**Table S2**. List of primers used in this study.
**Table S3.** Overview of the arbitrary PCR programs used in identification of the transposon insertion site.
**Figure S1.** Complete alignment of the amino acid sequences of the LapD homologs from *Pseudomonas putida* KT2440 (PP0165), *Pseudomonas fluorescens* Pf0‐1 (Pfl01_0131), and *Pseudomonas aeruginosa* PAO1 (PA1433). Black and gray shading denotes identical and similar residues, respectively, across all three sequences. ^Degenerate GGDEF and EAL motifs. #Functionally important residues as described by Newell et al. (2009), and Navarro et al. (2011).
**Figure S2.** Complete alignment of the amino acid sequences of the LapG homologs from *Pseudomonas putida* KT2440 (PP0164), *Pseudomonas fluorescens* Pf0‐1 (Pfl01_0130), and *Pseudomonas aeruginosa* PAO1 (PA1434). Black and gray shading denotes identical and similar residues, respectively, across all three sequences. ^The catalytic triad as predicted by Ginalski et al. (2004). #Functionally important calcium binding residues as described by Boyd et al. (2014).
**Figure S3.** Aggregation phenotype of transposon mutants in liquid cultures. Images are of outgrown cultures in glass tubes.
