# Supplementary material for: The LapG protein plays a role in Pseudomonas aeruginosa biofilm formation by controlling the presence of the CdrA adhesin on the cell surface
Source: Microbiologyopen. 2015 Oct 12;4(6):917–30. doi: 10.1002/mbo3.301 (PMC4694147; doi:10.1002/mbo3.301)
Supplement: Supplementary file 1 — Table S1. Full list of strains and plasmids used in this study. Table S2. List of primers used in this study. Table S3. Overview of the arbitrary PCR programs used in identification of the transposon insertion site. Figure S1. Complete alignment of the amino acid sequences of the LapD homologs from Pseudomonas putida KT2440 (PP0165), Pseudomonas fluorescens Pf0‐1 (Pfl01_0131), and Pseudomonas aeruginosa PAO1 (PA1433). Black and gray shading denotes identical and similar residues, respectively, across all three sequences. ^Degenerate GGDEF and EAL motifs. #Functionally important residues as described by Newell et al. (2009), and Navarro et al. (2011). Figure S2. Complete alignment of the amino acid sequences of the LapG homologs from Pseudomonas putida KT2440 (PP0164), Pseudomonas fluorescens Pf0‐1 (Pfl01_0130), and Pseudomonas aeruginosa PAO1 (PA1434). Black and gray shading denotes identical and similar residues, respectively, across all three sequences. ^The catalytic triad as predicted by Ginalski et al. (2004). #Functionally important calcium binding residues as described by Boyd et al. (2014). Figure S3. Aggregation phenotype of transposon mutants in liquid cultures. Images are of outgrown cultures in glass tubes. [file MBO3-4-0917-s001.docx]

**Supplementary information**

**The LapG protein plays a role in *Pseudomonas aeruginosa* biofilm formation by controlling the presence of the CdrA adhesin on the cell surface**

Morten Rybtke, Jens Berthelsen, Liang Yang, Niels Høiby, Michael Givskov, and Tim Tolker-Nielsen

**Table S1. Full list of strains and plasmids used in this study.**

| **Strain or plasmid** | **Relevant genotype and/or characteristics** | **Reference or source^a^** |
| --- | --- | --- |
| Strains |  |  |
| *P. aeruginosa* PAO1 |  |  |
| Wild-type |  | Stover *et al.*, 2000 |
| *lapG* | *lapG* deletion mutant | This study |
| *lapG cdrA* | *lapG cdrA* double deletion mutant | This study |
| *pel* | *pelA* deletion mutant | Rybtke *et al.*, 2012 |
| *pel lapG* | *pelA* and *lapG* double deletion mutant | This study |
| *psl* | *pslBCD* deletion mutant | This study |
| *psl lapG* | *pslBCD* and *lapG* double deletion mutant | This study |
| *wspF* | *wspF* deletion mutant | Rybtke *et al.*, 2012 |
| *wspF lapG* | *wspF* and *lapG* double deletion mutant | This study |
| *wspF cdrA* | *wspF* and *cdrA* double deletion mutant | This study |
| *wspF lapG cdrA* | *wspF*, *cdrA* and *lapG* triple deletion mutant | This study |
| *wspF pel* | *wspF* and *pelA* double deletion mutant | Rybtke *et al.*, 2012 |
| *wspF pel lapG* | *wspF*, *pelA*, and *lapG* deletion mutant | This study |
| *wspF psl* | *wspF* and *pslBCD* deletion mutant | This study |
| *wspF psl lapG* | *wspF*, *pslBCD*, and *lapG* triple deletion mutant | This study |
| *wspF pel psl* | *wspF*, *pelA*, and *pslBCD* triple deletion mutant | Rybtke et al., 2012 |
| *wspF pel psl lapG* | *wspF*, *pelA*, *pslBCD, and lapG* quadruple deletion mutant | This study |
| *wspF pel psl cdrA* | *wspF*, *pelA*, *pslBCD, and cdrA* quadruple deletion mutant | This study |
| *P. putida* OUS82 |  |  |
| Wild-type |  | Kiyohara *et al.*, 1994 |
| *lapD* | *lapD* deletion mutant | Gjermansen *et al.*, 2005 |
| *lapG* | *lapG* deletion mutant | Gjermansen *et al.*, 2005 |
| *E. coli* |  |  |
| DH5α | Classical cloning strain | Lab collection |
| MS690 | MG1655 ΔhsdR cloning strain | Lab collection |
| S17-1 λpir | Classical cloning and conjugation strain | Lab collection |
| SM10 λpir | Classical cloning and conjugation strain | Lab collection |
| Plasmids |  |  |
| Knock-out vectors |  |  |
| pΔlapG | *lapG* deletion vector, Gm^R^ | This study |
| pΔcdrA | *cdrA* deletion vector, Gm^R^ | This study |
| pMPSL-KO1 | *pslBCD* deletion vector, Amp^R^/Carb^R^ Gm^R^ | Kirisits *et al.*, 2005 |
| Expression vectors |  |  |
| pBBR1MCS-5 | Broad-host-range expression vector, Gm^R^ | Kovach *et al.*, 1995 |
| pPA1433 | pBBR1MCS-5-based *lapD* expression vector, Gm^R^ | This study |
| pPA1434 | pBBR1MCS-5-based *lapG* expression vector, Gm^R^ | This study |
| pMJT-1 | pUCP18-based araC-P_BAD_ expression vector, Amp^R^/Carb^R^ | Kaneko *et al*., 2007 |
| pBADcdrAB | pMJT-1-based P. aeruginosa PAO1 cdrAB expression vector, Amp^R^/Carb^R^ | Borlee *et al*., 2010 |
| Cloning vectors |  |  |
| pDONRPEX18Gm | Vector used in creating the knock-out/-in vectors of this study, Gm^R^ | Joe J. Harrison |
| Helper vectors |  |  |
| pRK600 | Mobilization vector for Tn7-tagging of *Pseudomonas* strains, Cm^R^ | Kessler *et al.*, 1992 |
| Misc. vectors |  |  |
| pBT20 | TnMariner delivery vector for transposon mutagenesis, Gm^R^ Cm^R^ | Kulasekara *et al.*, 2005 |
| pFlp2 | Delivery vector for Flp-mediated excision of FRT-flanked chromosomal insertions, Amp^R^/Carb^R^ | Hoang *et al.*, 1998 |
| ^a^A reference list is included at the end of this document. | | |

**Table S2. List of primers used in this study.**

| **Name** | **Sequence (5’ - 3’)** |
| --- | --- |
| Deletion vector construction |  |
| pΔlapG |  |
| PA1434_UpF | GGGGACAAGTTTGTACAAAAAAGCAGGCTCAGTCATCGATGGTCGCCAG |
| PA1434_UpR | TGACATTCAATCCTCCTTGGGTGCCAGCAGCAATGTCGC |
| PA1434_DnF | CCCAAGGAGGATTGAATGTCA |
| PA1434_DnR | GGGGACCACTTTGTACAAGAAAGCTGGGTAGAATTGCGGATGGCTGACC |
| pΔcdrA |  |
| cdrA_upF | GGGGACAAGTTTGTACAAAAAAGCAGGCTCATCTTGCCTTCCAGTTCGAC |
| cdrA_upR | CCAGGTAGCTCTGGTTCGACCCTTCCCTACCAGCTTTC |
| cdrA_dnF | TCGAACCAGAGCTACCTGG |
| cdrA_dnR | GGGGACCACTTTGTACAAGAAAGCTGGGTAGATCTGCTCGTAGAAGCCC |
| Knockout/-in verification |  |
| *lapG* |  |
| PA1434_seqF | GCATACAGGCTGCGGTAG |
| PA1434_seqR | TCCACCATCTGGTTCATCG |
| *cdrA* |  |
| cdrA_seqF | CAACTGACCGAGCAGCAC |
| cdrA_seqR | CCATGATCTGCAGGGTATC |
| *pslBCD* |  |
| pslBCD-Up | GCTGTTCCGCACCCTGGACGACT |
| pslBCD-Dn | TGCCGAACGCCGTGGTGA |
| Expression vector construction |  |
| pPA1433 |  |
| PA1433_fwd | TTTAAGCTTCTAGTGGAGGAATTCGATGTCACTGCTCAAGCAATTGT |
| PA1433_rev | AAATCTAGAATGCCGAATCCGCTGTAAAG |
| pPA1434 |  |
| PA1434_few | TTTAAGCTTCTAGTGGAGGAATTCGATGTCCCCAACCCCC |
| PA1434_rev | AAATCTAGATCAATCCTCCTTGGGTGC |
| Transposon mutagenesis |  |
| Arbitrary PCR round 1 |  |
| Rnd1-Pp1 | GGC CAC GCG TCG ACT AGT CAN NNN NNN NNN GAT AT |
| Rnd1-Pp2 | GGC CAC GCG TCG ACT AGT CAN NNN NNN NNN ACG CC |
| Rnd1-TnM | GTG AGC GGA TAA CAA TTT CAC ACA G |
| Arbitrary PCR round 2 |  |
| Rnd2-Pp | GGC CAC GCG TCG ACT AGT CA |
| Rnd2-TnM | ACA GGA AAC AGG ACT CTA GAG G |
| Sequencing |  |
| TnMseq | CAC CCA GCT TTC TTG TAC AC |

**Table S3. Overview of the arbitrary PCR programs used in identification of the transposon insertion site.**

| **Program** |  |  |  |  |  |  |
| --- | --- | --- | --- | --- | --- | --- |
| *Round 1* |  |  |  |  |  |  |
|  | 95°C 30'' |  | 94°C 30'' |  |  |  |
| 95°C 5' | 30°C 30'' | 6x | 45°C 1' | 30x | 72°C 7' | 4°C ∞ |
|  | 72°C 1' |  | 72°C 2' |  |  |  |
|  |  |  |  |  |  |  |
| *Round 2* |  |  |  |  |  |  |
|  | 95°C 30'' |  |  |  |  |  |
| 95°C 5' | 30°C 30'' | 30x | 72°C 7' | 4°C ∞ |  |  |
|  | 72°C 1' |  |  |  |  |  |
|  |  |  |  |  |  |  |

**Figure S1. Complete alignment of the amino acid sequences of the LapD homologs from *P. putida* KT2440 (PP0165), *P. fluorescens* Pf0-1 (Pfl01_0131), and *P. aeruginosa* PAO1 (PA1433).** Black and grey shading denotes identical and similar residues, respectively, across all three sequences. ^ Degenerate GGDEF and EAL motifs. # Functionally important residues as described by Newell *et al.* (2009), and Navarro *et al.* (2011).

KT2440_LapD 1 MSLFKQLLLAICLFLVVAFSGSFMVSLESSRSQYVNQLRSHAQDAATALALSLTPNIDDP
Pf0-1_LapD 1 MSLFKQLLIAICLFLVVAFTGSFMVSLESSRTQYVNQLRSHAQDAATALALSLTPNIDDP
PAO1_PA1433 1 MSLLKQLFLAICLFLVVAFSGSFVSSVENSREQLRGQLRSHAQDAATALGLSLTPHVDDP


KT2440_LapD 61 AMVELMVSSIFDSGYYASIKVVDLGSNAVLVERHAEPDPGGVPLWFVRLIGLEAAGGDAI
Pf0-1_LapD 61 AMVELLVSSIFDSGYYSSIRVVDLKTDQTIVERNGIPAVTNVPDWFVKLIGLEPAGGDAL
PAO1_PA1433 61 AMVQLMVSSIFDSGYFASIRVIDIKSGKPLVERVQAHAERTVPGWFERLVDLQPQGGDAL

 # #
KT2440_LapD 121 VSRGWQQAARVEVISHPMFAIAKLWQSALGSLGWLLLCGAASAVLGALLLRRQLRPLDYM
Pf0-1_LapD 121 VSRGWEQAARVEVVSHPMFALAKLWQSALGSLGWLLVCGAVSAVLGALLLRRQLKPLDYM
PAO1_PA1433 121 IMRGWEQAARVEVVSHPQFALARLWDSALGSLYWLLACGAASLLLGGWLLRRQLRPLDQM


KT2440_LapD 181 VEQSHAIARREFLSLPELPRTPELRRVVQAMNQMVEKLKALFTEQAERSERLRAESYQDS
Pf0-1_LapD 181 VKQSHAIARREFLSLPDLPRTPELRRVVLAMNQMVEKLKALFQEQAERSEKLRTESYQDN
PAO1_PA1433 181 VRQAHAISRREFLSLPRLPRTPELRRVVQAMNQMVEKLRTLFAEEAARSDKLRAQAYQDS


KT2440_LapD 241 LTGLSNRRYFEMQLNNRVSNLEDARAGYLLLLRVQGLAGLNARLGGQRTDQLLQAVGEQL
Pf0-1_LapD 241 LTGLANRRYFEMQLNARVSNPEQASSGYLLLLRVKDLAGLNQRLGGQRTDELLKAVGEQL
PAO1_PA1433 241 LTGLPNRRLFDARLNEQLGAGEHEHAGQLLLLRLNDLNGLNQRLGGQRTDELIQAVARLL

 ^^^^^
KT2440_LapD 301 RRTCASYPETNDLISRSRGGEFAVLAPGMVHEEAVHLAQALEATLQSLHETGASDIDPVA
Pf0-1_LapD 301 SRECAKYPETQNLVTRIRGGEFAVLAPGMTREEALQLAQSLDSALSSLYATGATDVAAVA
PAO1_PA1433 301 VDSCGQQGRADWLLARSRGGEFAVLAPGCSREQAERLAEELCEGLENLARTGASDLTPVA


KT2440_LapD 361 CIGLAPFSPGDSPQALLKLADEALARAENQPTPGWVCLEQGVAAVAADSQHAWHERLDQA
Pf0-1_LapD 361 SIGLAPFAHGDSPQAVLSLGDQALAQAEGQGEQNWACLDQSLVADVGDDHHAWHRLLDQA
PAO1_PA1433 361 YLGISAFAEGDSPADLLARADQALAQAESQPAQPWASQDGTALAALNDSQ-DWHDWIDQA

 ^^^ #
KT2440_LapD 421 FINGHFELFFQPVIECASSQRVLHHKVISRLRDGQGEALPAGRFLPWLERFGWMPRLDVL
Pf0-1_LapD 421 LNQRRFELFFQPVVAAQDTQLVLHYKVLSRLLDEQGQTIPAGRFLPWLERFGWTARLDRL
PAO1_PA1433 420 LTERRLLLYFQPVVDCLDTQRVLHHKVLARLLDPQATAIAAGRFLPWIERFGWAARMDLA


KT2440_LapD 481 VLEKVLAHLRGHDQVLALNLSAATLADPKALQRVFELLGQNAALGPRLVFEIGEEQLPEQ
Pf0-1_LapD 481 MLERVLEQMAGHEESLALNLSSATLADPQALNKVFEILRAHSNLGARLTLEIGEEQLPEQ
PAO1_PA1433 480 MLEQSLEHLRRHPRPLALSLSAASVRNAQTFAPLLALLKAHPQEARQLTLELDERHLPAA

 # # #
KT2440_LapD 541 AALEQLTRRLHGLGFGLALQRFGGRFSMIGNLAHLGLAYLKIDGSYIRNIDHEQHKRLFI
Pf0-1_LapD 541 AVLEQLTRRLRELGFSLSLQRFGGRFSMIGNLARLGLAYLKIDGSYIRAIDQESDKRLFI
PAO1_PA1433 540 AELERLSQVLRELGCGLGLQHFGGRFSLIGNLTHLGLAYLKLDGCYLHAVDREGDKRLFI

 # #
KT2440_LapD 601 EAIQRAAHSIDLPLIAERVETEGERLVLLEMGVGGIQGQLVGEPAPWR---
Pf0-1_LapD 601 EAIQRAAHSIDLPLIAERVETEGELSVIREMGLYGVQGQLFGEPKPWG---
PAO1_PA1433 600 EAVYRTTHSIDLPLIAEQVETLGELEVLREMGLRGAMGRLFGSPAPWSGDA

**Figure S2. Complete alignment of the amino acid sequences of the LapG homologs from *P. putida* KT2440 (PP0164), *P. fluorescens* Pf0-1 (Pfl01_0130), and *P. aeruginosa* PAO1 (PA1434).** Black and grey shading denotes identical and similar residues, respectively, across all three sequences. ^ The catalytic triad as predicted by Ginalski *et al.* (2004). # Functionally important calcium binding residues as described by Boyd *et al.* (2014).

KT2440_LapG 1 --------------------------------------MLLGSLLLGGLHADWDFSQISR
Pf0-1_LapG 1 MFFAGLTFTIKRFLLCPGCLALAVRFAIPRIARWLACALLLAGIMLGGLHADWDFSAISR
PAO1_PA1434 1 ------------MSPTPGARRPCPRAYAPWLLS-LAATLLLA---VGAALAQWDLESILS

KT2440_LapG 23 KSQALYGPLGAGQGRIDAWQSLMATQKQGTELERLQVVNRFFNQQLRYVEDIDLWHEVDY
Pf0-1_LapG 61 KATALYGPLGAGQQRIDAWQNLLATQKQVSEMEKLKVVNLFFNKQMRYVEDIDLWHEVDY
PAO1_PA1434 45 RAEQRYGELGAAKSRLGDWGRLLEQGGTLDEAAKLRAVNDFFNRSLRFTDDIEIWQQEDY

 ^#^ #
KT2440_LapG 83 WATPVQALIKGAGDCEDYAIAKYFSLRRMGIPSEKLRITYVKALRQNRAHMVLTYYSSPQ
Pf0-1_LapG 121 WETPIEALWKGAGDCEDYAIAKYFSLRHLGVASDKLRITYVKALRQNRAHMVLTYYSSPD
PAO1_PA1434 105 WATPVEALVKGAADCEDYAIAKYVTLRRLGVASDKLRITYVKALRLNQAHMVLTWYASPG

 #
KT2440_LapG 143 AQPLVLDSLMDAIKPASQRTDLLPVYAFNGEGLWLTGAAGNKKVGDTKRLSRWQDLLKKM
Pf0-1_LapG 181 AMPLVLDSLIDPIKPAAERTDLLPVYSFNAEGLYLPGAKGNKKVGDTKRLSRWQDVLKKM
PAO1_PA1434 165 ADPLVLDNLIGEIRPASQRDDLLPVYAFNAEGLWLPGADGGRRTGDSKKLSRWQDLLTKM


KT2440_LapG 203 QAEGFPAEPVY--
Pf0-1_LapG 241 QAEGFPVETTN--
PAO1_PA1434 225 RAEGLDLDAPKED

**Figure S3. Aggregation phenotype of transposon mutants in liquid cultures.** Images are of outgrown cultures in glass tubes.

**
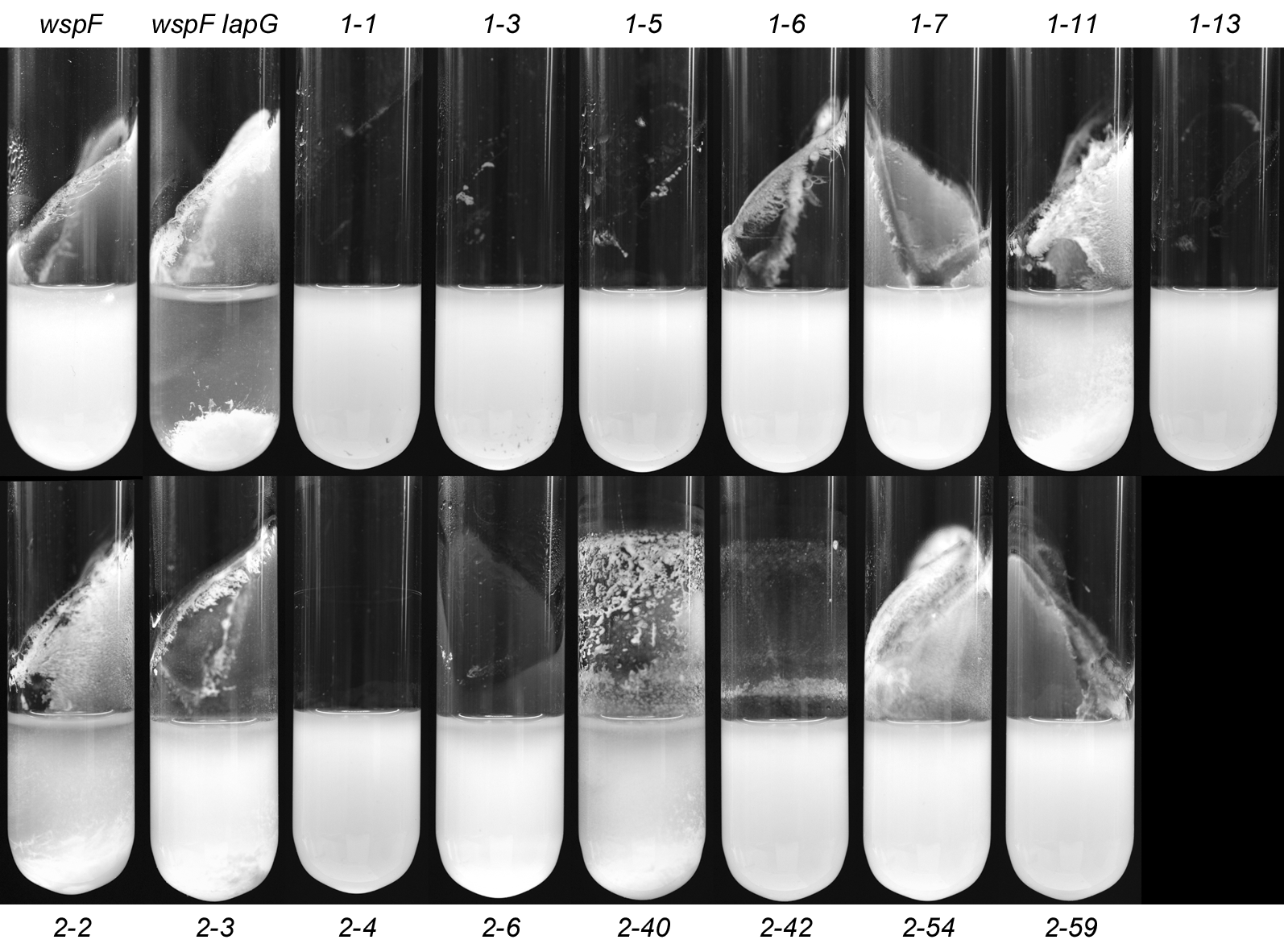
**

**References**

Borlee, B.R., Goldman, A.D., Murakami, K., Samudrala, R., Wozniak, D.J. & Parsek, M.R. (2010) *Pseudomonas aeruginosa* uses a cyclic-di-GMP-regulated adhesin to reinforce the biofilm extracellular matrix. *Mol Microbiol* **75**: 827-842.

Boyd, C.D., Smith, T.J., El-Kirat-Chatel, S., Newell, P.D., Dufrene, Y.F. & O'Toole, G.A. (2014) Structural features of the *Pseudomonas fluorescens* biofilm adhesin LapA required for LapG-dependent cleavage, biofilm formation, and cell surface localization. *J Bacteriol* **196**: 2775-2788.

Ginalski, K., Kinch, L., Rychlewski, L. & Grishin, N.V. (2004) BTLCP proteins: a novel family of bacterial transglutaminase-like cysteine proteinases. *Trends Biochem Sci* **29**: 392-395.

Gjermansen, M., Ragas, P., Sternberg, C., Molin, S. & Tolker-Nielsen, T. (2005) Characterization of starvation-induced dispersion in *Pseudomonas putida* biofilms. *Environ Microbiol* **7**: 894-906.

Hoang, T.T., Karkhoff-Schweizer, R.R., Kutchma, A.J. & Schweizer, H.P. (1998) A broad-host-range Flp-FRT recombination system for site-specific excision of chromosomally-located DNA sequences: application for isolation of unmarked *Pseudomonas aeruginosa* mutants. *Gene* **212**: 77-86.

Kaneko, Y., Thoendel, M., Olakanmi, O., Britigan, B.E. & Singh, P.K. (2007) The transition metal gallium disrupts Pseudomonas aeruginosa iron metabolism and has antimicrobial and antibiofilm activity. *J Clin Invest* **117**: 877-888.

Kessler, B., de Lorenzo, V. & Timmis, K.N. (1992) A general system to integrate lacZ fusions into the chromosomes of gram-negative eubacteria: regulation of the Pm promoter of the TOL plasmid studied with all controlling elements in monocopy. *Mol Gen Genet* **233**: 293-301.

Kirisits, M.J., Prost, L., Starkey, M. & Parsek, M.R. (2005) Characterization of colony morphology variants isolated from *Pseudomonas aeruginosa* biofilms. *Appl Environ Microbiol* **71**: 4809-4821.

Kiyohara, H., Torigoe, S., Kaida, N., Asaki, T., Iida, T., Hayashi, H. & Takizawa, N. (1994) Cloning and characterization of a chromosomal gene cluster, pah, that encodes the upper pathway for phenanthrene and naphthalene utilization by *Pseudomonas putida* OUS82. *J Bacteriol* **176**: 2439-2443.

Kovach, M.E., Elzer, P.H., Hill, D.S., Robertson, G.T., Farris, M.A., Roop, R.M., 2nd & Peterson, K.M. (1995) Four new derivatives of the broad-host-range cloning vector pBBR1MCS, carrying different antibiotic-resistance cassettes. *Gene* **166**: 175-176.

Kulasekara, H.D., Ventre, I., Kulasekara, B.R., Lazdunski, A., Filloux, A. & Lory, S. (2005) A novel two-component system controls the expression of *Pseudomonas aeruginosa* fimbrial cup genes. *Mol Microbiol* **55**: 368-380.

Navarro, M.V., Newell, P.D., Krasteva, P.V., Chatterjee, D., Madden, D.R., O'Toole, G.A. & Sondermann, H. (2011) Structural basis for c-di-GMP-mediated inside-out signaling controlling periplasmic proteolysis. *PLoS Biol* **9**: e1000588.

Newell, P.D., Monds, R.D. & O'Toole, G.A. (2009) LapD is a bis-(3',5')-cyclic dimeric GMP-binding protein that regulates surface attachment by *Pseudomonas fluorescens* Pf0-1. *Proc Natl Acad Sci U S A* **106**: 3461-3466.

Rybtke, M.T., Borlee, B.R., Murakami, K., Irie, Y., Hentzer, M., Nielsen, T.E.*, et al.* (2012) Fluorescence-based reporter for gauging cyclic di-GMP levels in *Pseudomonas aeruginosa*. *Appl Environ Microbiol* **78**: 5060-5069.

Stover, C.K., Pham, X.Q., Erwin, A.L., Mizoguchi, S.D., Warrener, P., Hickey, M.J.*, et al.* (2000) Complete genome sequence of *Pseudomonas aeruginosa* PAO1, an opportunistic pathogen. *Nature* **406**: 959-964.
